# The Association Between Embryo Quality, Number of Transferred Embryos and Live Birth Rate After Vitrified Cleavage-Stage Embryos and Blastocyst Transfer

**DOI:** 10.3389/fphys.2020.00930

**Published:** 2020-08-14

**Authors:** Qianqian Zhu, Jiaying Lin, Haoyuan Gao, Ningling Wang, Bian Wang, Yun Wang

**Affiliations:** Department of Assisted Reproduction, Shanghai Ninth People’s Hospital Affiliated to Jiao Tong University School of Medicine, Shanghai, China

**Keywords:** embryo quality, number of embryo transfer, live birth rate, vitrification, frozen embryo transfer

## Abstract

**Objective:**

The single-embryo transfer (SET) is the recommended approach to improve the live birth rate and reduce the complications related with multiple pregnancies. However, the physicians generally chose to transfer two embryos when the embryo quality decreased. The effect on the *in vitro* fertilization (IVF) or intracytoplasmic sperm injection (ICSI) outcomes following the transfer of a poor-quality embryo (PQE) along with a good-quality embryo (GQE) has been explored. However, previous studies were limited by the fresh embryo transfer cycles or the small sample size.

**Methods:**

A retrospective cohort study was performed among 26,676 women (the mean age was 31.72 years) undergoing first frozen embryo transfer (FET) from January 2011 to December 2017. Patients were grouped into five subgroups, including SET with one GQE (SET-GQE, 2235 patients for cleavage-stage embryo transfer and 756 patients for blastocyst transfer), SET with one PQE (SET-PQE, 148 patients for cleavage-stage embryo transfer and 362 patients for blastocyst transfer), double-embryo transfer with two GQE (DET-2GQE, 20,461 patients for cleavage-stage embryo transfer and 519 patients for blastocyst transfer), double-embryo transfer (DET) with one GQE plus one PQE (DET-GQE+PQE, 1541 patients for cleavage-stage embryo transfer and 266 patients for blastocyst transfer), and DET with two PQE (DET-2PQE, 228 patients for cleavage-stage embryo transfer and 160 patients for blastocyst transfer). Multivariable logistic regression models were performed after controlling for other potential confounders to estimate the effect of number and quality of transferred embryos on pregnancy outcomes.

**Result:**

Although the live birth rate was significantly higher after DET-GQE+PQE compared with SET-GQE for cleavage-stage embryo transfer [574 of 1541 (37.25%) vs. 571 of 2235 (25.55%)], no significant difference was found between DET-GQE+PQE and SET-GQE for blastocyst transfer [143 of 266 (53.76%) vs. 325 of 756 (42.99%)]. However, DET-GQE+PQE also had the highest multiple live births in both cleavage-stage embryo transfer [134 of 1541 (8.70%)] and blastocyst transfer [46 of 266 (17.29%)]. The live birth rate after SET-PQE significantly decreased in comparison with SET-GQE [cleavage-stage embryo transfer: 18 of 148 (12.16%) vs. 571 of 2235 (25.55%); blastocyst transfer: 107 of 362 (29.56%) vs. 325 of 756 (42.99%)] and significantly increased after DET-2GQE compared with SET-GQE [cleavage-stage embryo transfer: 9357 of 20,461 (45.73%) vs. 571 of 2235 (25.55%); blastocyst transfer: 313 of 519 (60.31%) vs. 325 of 756 (42.99%)]. The live birth rate was also not different between DET-2PQE and SET-GQE for cleavage-stage embryo transfer and blastocyst transfer [cleavage-stage embryo transfer: 75 of 228 (32.89%) vs. 571 of 2235 (25.55%); blastocyst transfer: 74 of 160 (46.25%) vs. 325 of 756 (42.99)].

**Conclusion:**

In order to minimize the risk of multiple births, the data from this study did not support transferring DET with a GQE plus a PQE compared with SET with a GQE, especially for blastocyst transfer. However, the proportion of patients older than 35 years was small (12.07% for patients aged 36–39 years and 7.31% for patients 40 years or older), which limited the generalization of these results to other population.

## Introduction

The development of clinical and laboratory techniques over decades has not only improved the live birth rate after assisted reproduction technology (ART) but also increased the multiple pregnancy rate, which increased the obstetric complication and adverse neonatal outcomes (Neubourg et al., 2002; McLernon et al., 2010). As the ultimate goal of ART is the birth of a healthy child with no maternal complication, the single-embryo transfer (SET) is the recommended approach to achieve this goal and reduce the complications related with multiple pregnancies. However, embryo quality is one important factor to consider when implementing a successful SET. Embryo quality, based on morphological parameters, is a major predictor for the success of implantation and live birth (Della et al., 2007; Ahlstrom et al., 2011). The study conducted by Oron et al. (2014) reported the clinical pregnancy rate and live birth rate after the single good-quality embryo transfer (GQE) were almost twice as high as those after the single poor-quality embryo (PQE) transfer.

Despite the higher multiple pregnancy rate, the physicians generally chose to transfer two embryos when the embryo quality decreased. Two studies have explored the effect on the *in vitro* fertilization (IVF) or intracytoplasmic sperm injection (ICSI) outcomes following the transfer of a PQE along with a GQE and found that the clinical pregnancy rate and live birth rate were similar when compared with transfer of two GQEs, but the above studies were all conducted in the fresh cycles (Wintner et al., 2017; Li et al., 2018). Furthermore, Dobson et al. (2018) evaluated the influence of double-embryo transfer (DET) with a PQE plus a GQE on the IVF outcome in the fresh and frozen cycles and reported that the live birth rate did not increase but the multiple birth increased compared with SET. However, this study only included blastocyst transfer and the sample size was small in five subgroups divided by the number and quality of embryos available. Therefore, the impact of DET and SET on IVF outcome at different grades of embryo quality in the vitrified cleavage and blastocyst stages needed to be further explored in a large sample study.

We conducted this research aiming at exploring the differences in the association between pregnancy outcomes and number of transferred embryos by grades of embryo quality in both vitrified cleavage-stage embryo transfer and blastocyst transfer.

## Materials and Methods

### Study Population

In this retrospective cohort study, we included 26,676 women receiving their first frozen embryo transfer (FET) in the time period from January 2011 to December 2017 at the Department of Assisted Reproduction of the Shanghai Ninth People’s Hospital affiliated to Jiao Tong University School of Medicine (a large hospital-based tertiary care reproductive center in Shanghai, China). All included women used autologous oocytes, and each woman was included only once in this study. Women with previous fresh or FET were excluded. This study protocol was approved by the Ethics Committee (Institutional Review Board) of the Shanghai Ninth People’s Hospital.

The details about ovulation induction and IVF/ICSI procedure, embryo culture and evaluation, freezing and thawing of embryos, and FET all have been detailed in our previous articles (Kuang et al., 2014a, b; Chen et al., 2015; Du et al., 2017). IVF or ICSI was performed depending on the semen quality. Normal fertilization was assessed 16–18 h after insemination/injection. Then, the embryos were subsequently cultured until day 3 or day 5/6.

Cleavage embryos were classified as GQEs (grade I and II embryos) if they had four cells on day 2 or six to eight cells on day 3, with less than 20% anucleate fragments according to Cummins criteria (Cummins et al., 1986; Reinblatt et al., 2011). Embryos graded III or IV including those that had only two cells on day 2, less than six cells on day 3, and no less than 20% fragmentation were called poor quality. Blastocyst quality was graded on day 5 or 6 according to the degree of blastocoel expansion, inner cell mass (ICM), and trophectoderm (TE) cells (Gardner et al., 2004). Good-quality embryos were defined as those where at least (3) the blastoceles were filling completely 100% of the embryo, (B) they are loosely grouped with several cells, and (B) several cells formed in the loose epithelium. Lower-than-3BB-quality embryos on day 5 or 6 were defined as PQEs. The allocation to blastocyst- or cleavage-stage embryo transfer depends on the patient’s age and the quality and number of cleavage-stage embryos available. Embryo grading was done by two trained embryologists and was verified by another senior embryologist with years of work experience.

After embryo grading, all cleavage-stage embryos and blastocysts were frozen using the vitrification method. In brief, the cryotop carrier system (Kitazato Biopharma Co., Ltd., Japan) was used for vitrification and 15% (v/v) ethylene glycol, 15% (v/v) dimethyl sulfoxide, and 0.5 mol/l sucrose were used as the cryoprotectant. For warming, 1.0, 0.5, and 0.0 mol/l sucrose solutions were used for stepwise cryoprotectant dilution. All vitrification and warming steps were carried out at room temperature except the first warming step, which was at 37°C. The same vitrification method was employed throughout the whole study period.

Endometrial preparation was performed as previously described (Du et al., 2017). Natural cycle was used for patients with regular menstrual cycles, and hormone therapy cycle or stimulation cycle was used for patients with irregular menstrual cycles. One or two embryos were transferred, and P supplementation was provided until 8 weeks of gestation if pregnant. Patients were excluded if they had mixed-cleavage–blastocyst-stage embryo transfer.

A live birth was defined as an infant born alive after 24 weeks of gestation who survived more than 28 days. The patients were grouped into cleavage-stage embryo group and blastocyst group according to the development stage of transferred embryos. Then, for each group, five subgroups were formed according to the number and quality of embryos, SET-GQE included patients with single GQE transfer, SET-PQE consisted of patients with single PQE transfer, DET-2GQE involved patients with double GQE transfer, DET-GQE+PQE were those patients with one GQE and one PQE transfer, and DET-2PQE were patients with two PQEs transfer.

### Statistical Analysis

The baseline characteristics were described with mean (standard deviation, SD) for continuous variables and percentage for categorical variables. Comparisons of baseline characteristics between groups were performed with chi-square test or ANOVA where appropriate. The live birth rates across subgroups stratifying by stage of embryo development were calculated. Multivariable logistic regression was performed to explore the effect of different embryo transfer strategies on the live birth for cleavage-stage embryo transfer and blastocyst transfer, respectively, after controlling for potential confounders including maternal age, maternal BMI, infertility type, parity, duration of infertility, causes of infertility, number of two pronuclear (2PN) embryos, endometrial preparation protocol, and endometrial thickness. Results were reported as adjusted odds ratios (aORs) with 95% confidence intervals (CIs). All statistical analyses were performed by using the two-sided 5% level of significance and the statistical package Stata, Version 12 (StataCorp, College Station, TX, United States).

## Results

In total, 26,676 patients underwent the first FET (24,613 patients with cleavage-stage embryo transfer and 2063 with blastocysts transfer) in the study period from 2011 to 2017. Among all patients, 2991 patients underwent transfer of single GQE, 510 patients underwent single PQE, 20,980 patients underwent double GQEs, 1807 patients had DET with one GQE plus one PQE, and 388 patients had two PQEs transfer. The patient and treatment characteristics are presented in Table 1. The proportion of patients less than or equal to 30 years of age (46.68%) was higher in the group with double GQE transfer, and the proportion of patients 35 years of age or greater (37.06%) was higher in the group with single PQE transfer. More than 80% of patients were nulliparous, and the major infertility cause was female factor.

**TABLE 1:** Maternal and treatment characteristics by group of embryo transfer.

**Demographics**	**SET-GQE (*n* = 2991)**	**SET-PQE (*n* = 510)**	**DET-2GQE (*n* = 20,980)**	**DET-GQE+PQE (*n* = 1807)**	**DET-2PQE (*n* = 388)**	***P*-value**
Patients with eSET or eDET	1266 (42.33)	170 (33.33)	16,135(76.91)	971 (53.74)	195 (50.26)	
Maternal age(y), *n* (%)						<0.001
≤30	1080 (36.11)	157 (30.78)	9793 (46.68)	686 (37.96)	144 (37.11)	
31–34	866 (28.95)	164 (32.16)	6521 (31.08)	553 (30.60)	117 (30.15)	
≥35	1045 (34.94)	189 (37.06)	4666 (22.24)	568 (31.43)	127 (32.73)	
Type of infertility, *n* (%)						0.271
Primary infertility	1599 (53.46)	264 (51.76)	11,370(54.19)	958 (53.02)	193 (49.74)	
Second infertility	1392 (46.54)	246 (48.24)	9610 (45.81)	849 (46.98)	195 (50.26)	
Parity, *n* (%)						<0.001
Nulliparous	2577 (86.16)	421 (82.55)	18,862(89.90)	1568 (86.77)	330 (85.05)	
Pluriparous	414 (13.84)	89 (17.45)	2118 (10.10)	239 (13.23)	58 (14.95)	
Duration of infertility, mean ± SD	3.95 ± 3.07	4.09 ± 3.10	3.72 ± 2.71	4.02 ± 2.91	4.20 ± 3.02	<0.001
Infertility causes, *n* (%)						0.008
Female factor	1816 (60.72)	294 (57.65)	12,725(60.65)	1054 (58.33)	241 (62.11)	
Male factor	282 (9.43)	49 (9.61)	2281 (10.87)	194 (10.74)	44 (11.34)	
Combined factor	631 (21.10)	102 (20.00)	4219 (20.11)	377 (20.86)	70 (18.04)	
Unexplained	262 (8.76)	65 (12.75)	1755 (8.37)	182 (10.07)	33 (8.51)	
Stage of embryo development						<0.001
Cleavage-stage embryo	2235 (74.72)	148 (29.02)	20,461(97.53)	1541 (85.28)	228 (58.76)	
Blastocyst	756 (25.28)	362 (70.98)	519 (2.47)	266 (14.72)	160 (41.24)	

*SET, single embryo transfer; DET, double embryos transfer; GQE, good quality embryo; PQE, poor quality embryo; FET: frozen-thawed embryo transfer; eSET, elective SET; eDET, elective DET.*

In general, transfer of blastocyst had a significantly higher rate of live birth than did transfer of cleavage-stage embryo (for SET: blastocyst vs. cleavage-stage embryo 38.64% vs. 24.72%; for DET: blastocyst vs. cleavage-stage embryo 56.08% vs. 45.01%; Table 2). The multiple live birth rates were also significantly higher for blastocyst transfer compared with cleavage-stage embryo transfer (for SET: blastocyst vs. cleavage-stage embryo 0.81% vs. 0.29%; for DET: blastocyst vs. cleavage-stage embryo 21.69% vs. 13.76%).

**TABLE 2:** Live birth rate in the first FET cycles, stratified by number of embryo transfer, embryo quality, and stage of embryo development.

	**Cleavage-stage embryo transfer**			**Blastocyst transfer**		
	**Number of cycles**	**Live birth rate (%)**	**Multiple live birth rate (%)**	**Number of cycles**	**Live birth rate (%)**	**Multiple live birth rate (%)**
Total	24,613	43.05	12.45	2063	46.63	10.37
SET	2383	24.72	0.29	1118	38.64	0.81
SET-GQE	2235	25.55	0.31	756	42.99	0.79
SET-PQE	148	12.16	0	362	29.56	0.83
DET	22,230	45.01	13.76	945	56.08	21.69
DET-2GQE	20,461	45.73	14.22	519	60.31	26.20
DET-GQE+PQE	1541	37.25	8.70	266	53.76	17.29
DET-2PQE	228	32.89	6.14	160	46.25	14.37

*SET, single embryo transfer; DET, double embryos transfer; GQE, good quality embryo; PQE, poor quality embryo; FET: frozen-thawed embryo transfer.*

For patients with cleavage-stage embryo transfer, DET-2GQE had the highest live birth rate (45.73%), followed by DET-GQE+PQE (37.25%), DET-2PQE (32.89%), SET-GQE (25.55%), and SET-PQE which had the lowest live birth rate (12.16%). This trend also can be found among patients with blastocyst transfer. DET-2GQE also had the highest multiple live births in both cleavage-stage embryo transfer (14.22%) and blastocyst transfer (26.20%). In addition, the live birth rate was significantly higher after blastocyst transfer than after cleavage-stage embryo transfer for each of the five embryo transfer strategies.

Table 3 shows the results from multivariate logistic regression on the live birth rate following SET-GQE against SET-PQE, DET-2GQE, DET-GQE+PQE, and DET-2PQE for cleavage-stage embryo transfer and blastocyst transfer, respectively. For both cleavage-stage embryo transfer and blastocyst transfer, after adjusting for other confounders, the results showed that the live birth rate after SET-PQE significantly decreased in comparison with SET-GQE (cleavage-stage embryo transfer: aOR 0.49, 95% CI 0.28, 0.84; blastocyst transfer: aOR 0.62, 95% CI 0.46, 0.83), and live birth rate after DET-2GQE significantly increased compared with SET-GQE (cleavage-stage embryo transfer: aOR 1.62, 95% CI 1.40, 1.89; blastocyst transfer: aOR 1.76, 95% CI 1.20, 2.57). Although the live birth rate was significantly higher after DET-GQE+PQE compared with SET-GQE for cleavage-stage embryo transfer (aOR 1.25, 95% CI: 1.04, 1.51), no significant difference was found between DET-GQE+PQE and SET-GQE for blastocyst transfer (aOR 1.42, 95% CI: 0.93, 2.15). The live birth rate was also not different between DET-2PQE and SET-GQE for cleavage-stage embryo transfer and blastocyst transfer (cleavage-stage embryo transfer: aOR 1.10, 95% CI 0.78, 1.55; blastocyst transfer: aOR 1.09, 95% CI 0.67, 1.76).

**TABLE 3:** Logistic regression analysis for live birth rate after FET among patients with different embryo transfer strategies.

	**Unadjusted odds ratio (95% CI)**	***P*-value**	**Adjusted odds ratio (95% CI)**	***P*-value**
Cleavage-stage embryo transfer				
SET-GQE	1.00		1.00	
SET-PQE	0.40 (0.24,0.67)	<0.001	0.49 (0.28,0.84)	0.009
DET-2GQE	2.46 (2.22,2.71)	<0.001	1.62 (1.40,1.89)	<0.001
DET-GQE+PQE	1.73 (1.50,1.99)	<0.001	1.25 (1.04,1.51)	0.018
DET-2PQE	1.43 (1.07,1.92)	0.017	1.10 (0.78,1.55)	0.578
Blastocyst transfer				
SET-GQE	1.00		1.00	
SET-PQE	0.56 (0.43,0.73)	<0.001	0.62 (0.46,0.83)	0.001
DET-2GQE	2.01 (1.61,2.53)	<0.001	1.76 (1.20,2.57)	0.003
DET-GQE+PQE	1.54 (1.16,2.04)	0.003	1.42 (0.93,2.15)	0.101
DET-2PQE	1.14 (0.81,1.61)	0.450	1.09 (0.67,1.76)	0.726

*SET, single embryo transfer; DET, double embryos transfer; GQE, good quality embryo; PQE, poor quality embryo; FET: frozen-thawed embryo transfer. Adjusted maternal age, maternal BMI, infertility type, parity, duration of infertility, causes of infertility, number of two pronuclear (2PN) embryos, endometrial preparation protocol, and endometrial thickness.*

## Discussion

This large retrospective cohort study showed that DET with a GQE plus a PQE increased the live birth rate for cleavage-stage embryo transfer but did not increase the live birth rate for blastocyst transfer when compared with SET with a GQE during the first FET treatments. Furthermore, DET with a GQE plus a PQE increased multiple live births for both cleavage-stage embryo transfer and blastocyst transfer. Our study indicated that although DET with a GQE plus a PQE was helpful to improve the live birth rate, it led to the increasing risk of multiple live births for cleavage-stage embryo transfer, whereas DET with a GQE plus a PQE was not found to have any benefit in live birth rate, while increasing multiple births for blastocyst transfer. Therefore, our study advises to weigh up the pros and cons before making the clinical decisions about transferring DET with a GQE plus a PQE, especially for blastocyst transfer.

To our knowledge, this was one of the largest retrospective cohort studies exploring the live birth rate after DET with a GQE plus a PQE during the FET treatments. In this study, patients with both the cleavage-stage embryo transfer and blastocyst transfer were enrolled, the results of which could enrich current research in this field. In order to minimize the effect of confounding factors, we only included patients undergoing the first FET and adjusted variables including maternal age, maternal BMI, infertility type, parity, duration of infertility, causes of infertility, number of 2PN embryos, endometrial preparation protocol, and endometrial thickness. However, our study was limited by the retrospective design from the single center; the conclusion from our study needs to be verified in further studies. The proportion of patients older than 35 years was small (12.07% for patients aged 36–39 years and 7.31% for patients 40 years or older), which also limited the generalization of these results to other population. In addition, the majority of patients in this study underwent cleavage-stage embryo transfer rather than blastocyst transfer. The following reasons can account for the lower proportion of blastocyst transfer in our study. On the one hand, Chinese legislation limited the proportion of blastocyst transfer cycles to control the male birth, which leads to the transfer of cleavage-stage embryos remaining a priority in Chinese IVF centers. On the other hand, extending embryo culture to blastocyst from cleavage stage has been regarded as a tool of embryo selection. Although this procedure improves the live birth rate, it also increases the transfer cancelation rate and diminishes the number of viable embryos for cryopreservation and subsequent frozen-thawed embryo transfer. Previous study has found that the failure to reach the stage of embryo transfer because of poor or arrested embryo development increased in patients seeking blastocyst-stage embryo transfer compared with patients with cleavage-stage embryo transfer (Glujovsky et al., 2016). It has been reported that some cleavage-stage embryos that did not survive extended *in vitro* culture, however, may continue to develop into viable pregnancies if they were transferred into the uterus on day 3 (Gleicher et al., 2015). So many patients and clinicians choose to cleavage-stage embryo transfer after weighing the pros and cons.

There were different viewpoints on the effect of DET with mixed quality embryos on embryo development and growth. Some authors proposed that the implantation potential of each embryo was independent, and transferring a PQE would not affect the pregnancy and implantation of a GQE. However, the cooperative interaction was proposed to explain that preimplantation embryos could affect the surrounding embryo development by specific released growth factors (Tao et al., 2013). The interaction was related with the embryo quality, and factors from PQE could adversely impact the development of neighboring embryos. In recent years, the decidualized endometrial stromal cells (ESCs) have been a study focus, which could recognize GQE and PQEs and respond selectively to developmentally impaired embryos to prevent its implantation (Weimar et al., 2013). Many previous studies also evaluated the effect of DET with mixed quality embryos on live birth rate by clinical data, and the results were inconsistent. El-Danasouri et al. (2016) reported that transferring a morphologically impaired embryo significantly adversely affected the clinical pregnancy and implantation of the GQE in a retrospective multicenter study. Dobson et al. (2018) finished a prospective study and demonstrated that there exists a non-significant trend of decrease in live birth rate for DET with a GQE plus a PQE compared with SET with a GQE. They attributed the absence of significance to the small sample size and proposed that a PQE may detrimentally impact the implantation of a GQE for blastocyst transfer in both fresh and frozen cycles. Two studies performed in fresh IVF/ICSI cycles found that the live birth rate has no statistically significant differences for DET with mixed quality embryos vs. SET with a GQE or DET with two GQE (Wintner et al., 2017; Li et al., 2018). In contrast to previous studies, our study found the increased live birth rate after DET with a GQE plus a PQE compared with SET with a GQE for vitrified cleavage-stage embryo transfer, but no significant change was found in the live birth rate between DET with a GQE plus a PQE and SET with a GQE for blastocyst transfer. Given the conflicted results in the existing research, prospective randomized controlled trials were needed to confirm the relationship.

Consistent with previous studies, our research also showed that the multiple live birth rates per embryo transfer cycle were significantly increased after DET than SET regardless of the transferred embryo quality and the development stage of embryo (Gelbaya et al., 2010; McLernon et al., 2010). Since October 2015, the “universal two-child policy” has been enacted in China (Zeng and Hesketh, 2016; Li et al., 2019). Influenced by this policy, many infertile couples want to have two children with one ART treatment. However, multiple live births were associated with increased obstetrical and neonatal morbidity and mortality. From a healthcare and societal perspective, the cost was also found to be much higher for DET than cumulative SET (Kjellberg et al., 2006). McLernon et al. (2010) also proposed that the difference in live birth rate between SET and DET could be overcome through an additional SET. So we advise these infertile couples anticipating two children to undergo two separate childbirths after SET rather than DET.

In agreement with other studies, the present study found the significantly higher live birth rate after blastocyst transfer than cleavage embryo transfer (Zhu et al., 2013; Glujovsky et al., 2016). The likely explanation for this result was the embryo selection by extending culture to the blastocyst stage, which reduced the number of PQEs, as only those with strong developmental potential survive the challenge of extended culture and reach the blastocyst stage. In our research, the live birth rate for single blastocyst transfer with a GQE was higher than that for double cleavage-stage embryo transfer with mixed quality embryos and double cleavage-stage embryo transfer with two PQE. This result verified that a single blastocyst transfer with a GQE was the preferred recommendation for patients preparing for the first FET.

## Conclusion

In conclusion, DET with a GQE plus a PQE increased the live birth rate for cleavage-stage embryo transfer but did not increase the live birth rate for blastocyst transfer when compared with SET with a GQE during the first FET treatments. Furthermore, DET with a GQE plus a PQE increased multiple live births for both cleavage-stage embryo transfer and blastocyst transfer. Therefore, in order to minimize the risk of multiple births, the data from this study did not support transferring DET with a GQE plus a PQE compared with SET with a GQE, especially for blastocyst transfer.

## Data Availability Statement

The datasets generated for this study are available on request to the corresponding author.

## Ethics Statement

The studies involving human participants were reviewed and approved by the Ethics Committee (Institutional Review Board) of the Shanghai Ninth People’s Hospital. Written informed consent for participation was not required for this study in accordance with the national legislation and the institutional requirements.

## Author Contributions

QZ contributed to data collection and drafted the manuscript. HG, JL, NW, BW, and YW were responsible for the analysis of data. All authors have read and approved the final manuscript.

## Conflict of Interest

The authors declare that the research was conducted in the absence of any commercial or financial relationships that could be construed as a potential conflict of interest.

## References

[B1] Ahlstrom A. Westin C. Reismer E. Wikland M. Hardarson T. (2011). Trophectoderm morphology: an important parameter for predicting live birth after single blastocyst transfer. *Hum. Reprod.* 26 3289–3296. 10.1093/humrep/der325 21972253

[B2] Chen H. Wang Y. Lyu Q. Ai A. Fu Y. Tian H. (2015). Comparison of live-birth defects after luteal-phase ovarian stimulation vs. conventional ovarian stimulation for in vitro fertilization and vitrified embryo transfer cycles. *Fertil. Steril.* 103 1194.e2–1201.e2. 10.1016/j.fertnstert.2015.02.020 25813280

[B3] Cummins J. M. Breen T. M. Harrison K. L. Shaw J. M. Wilson L. M. Hennessey J. F. (1986). A formula for scoring human embryo growth rates in in vitro fertilization: its value in predicting pregnancy and in comparison with visual estimates of embryo quality. *J. In Vitro Fert. Embryo Transf.* 3 284–295. 10.1007/bf01133388 3783014

[B4] Della R. T. Verheyen G. Papanikolaou E. G. Van Landuyt L. Devroey P. Van Steirteghem A. (2007). Developmental stage on day-5 and fragmentation rate on day-3 can influence the implantation potential of top-quality blastocysts in IVF cycles with single embryo transfer. *Reprod. Biol. Endocrinol.* 5:2. 10.1186/1477-7827-5-2 17257401 PMC1796880

[B5] Dobson S. Lao M. T. Michael E. Varghese A. C. Jayaprakasan K. (2018). Effect of transfer of a poor quality embryo along with a top quality embryo on the outcome during fresh and frozen in vitro fertilization cycles. *Fertil. Steril.* 110 655–660. 10.1016/j.fertnstert.2018.05.010 30196962

[B6] Du T. Chen H. Fu R. Chen Q. Wang Y. Mol B. W. (2017). Comparison of ectopic pregnancy risk among transfers of embryos vitrified on day 3, day 5, and day 6. *Fertil. Steril.* 108 108–116. 28602476 10.1016/j.fertnstert.2017.05.027

[B7] El-Danasouri I. Sterzik K. Rinaldi L. Pacchiarotti A. DeSanto M. Selman H. (2016). Effect of transferring a morphologically impaired embryo with a good quality embryo on the pregnancy and implantation rates. *Eur. Rev. Med. Pharmacol. Sci.* 20 394–398. 26914111

[B8] Gardner D. K. Surrey E. Minjarez D. Leitz A. Stevens J. Schoolcraft W. B. (2004). Single blastocyst transfer: a prospective randomized trial. *Fertil. Steril.* 81 551–555. 10.1016/j.fertnstert.2003.07.023 15037401

[B9] Gelbaya T. A. Tsoumpou I. Nardo L. G. (2010). The likelihood of live birth and multiple birth after single versus double embryo transfer at the cleavage stage: a systematic review and meta-analysis. *Fertil. Steril.* 94 936–945. 10.1016/j.fertnstert.2009.04.003 19446809

[B10] Gleicher N. Kushnir V. A. Barad D. H. (2015). Is it time for a paradigm shift in understanding embryo selection? *Reprod. Biol. Endocrinol.* 13:3. 10.1186/1477-7827-13-3 25577140 PMC4326369

[B11] Glujovsky D. Farquhar C. Quinteiro R. A. Alvarez S. C. Blake D. (2016). Cleavage stage versus blastocyst stage embryo transfer in assisted reproductive technology. *Cochrane Database Syst. Rev.* 30:CD002118. 10.1002/14651858.CD002118.pub5 27357126

[B12] Kjellberg A. T. Carlsson P. Bergh C. (2006). Randomized single versus double embryo transfer: obstetric and paediatric outcome and a cost-effectiveness analysis. *Hum. Reprod.* 21 210–216. 10.1093/humrep/dei298 16172148

[B13] Kuang Y. Chen Q. Hong Q. Lyu Q. Ai A. Fu Y. (2014a). Double stimulations during the follicular and luteal phases of poor responders in IVF/ICSI programmes (Shanghai protocol). *Reprod. Biomed. Online* 29 684–691. 10.1016/j.rbmo.2014.08.009 25444501

[B14] Kuang Y. Hong Q. Chen Q. Lyu Q. Ai A. Fu Y. (2014b). Luteal-phase ovarian stimulation is feasible for producing competent oocytes in women undergoing in vitro fertilization/intracytoplasmic sperm injection treatment, with optimal pregnancy outcomes in frozen-thawed embryo transfer cycles. *Fertil. Steril.* 101 105–111. 10.1016/j.fertnstert.2013.09.007 24161646

[B15] Li H. T. Xue M. Hellerstein S. Cai Y. Gao Y. Zhang Y. (2019). Association of China’s universal two child policy with changes in births and birth related health factors: national, descriptive comparative study. *BMJ* 366:l4680. 10.1136/bmj.l4680 31434652 PMC6699592

[B16] Li J. Du M. Zhang Z. Guan Y. Wang X. Zhang X. (2018). Does a poor-quality embryo have an adverse impact on a good-quality embryo when transferred together? *J. Ovarian Res.* 11:78. 10.1186/s13048-018-0452-6 30180866 PMC6122748

[B17] McLernon D. J. Harrild K. Bergh C. Davies M. J. de Neubourg D. (2010). Clinical effectiveness of elective single versus double embryo transfer: meta-analysis of individual patient data from randomised trials. *BMJ* 341:c6945. 10.1136/bmj.c6945 21177530 PMC3006495

[B18] Neubourg D. D. Mangelschots K. Van Royen E. Vercruyssen M. Ryckaert G. Valkenburg M. (2002). Impact of patients’ choice for single embryo transfer of a top quality embryo versus double embryo transfer in the first IVF/ICSI cycle. *Hum. Reprod.* 17 2621–2625. 10.1093/humrep/17.10.2621 12351538

[B19] Oron G. Son W. Y. Buckett W. Tulandi T. Holzer H. (2014). The association between embryo quality and perinatal outcome of singletons born after single embryo transfers: a pilot study. *Hum. Reprod.* 29 1444–1451. 10.1093/humrep/deu079 24812313

[B20] Reinblatt S. L. Ishai L. Shehata F. Son W. Y. Tulandi T. Almog B. (2011). Effects of ovarian endometrioma on embryo quality. *Fertil. Steril.* 95 2700–2702. 10.1016/j.fertnstert.2011.03.002 21444070

[B21] Tao T. Robichaud A. Mercier J. Ouellette R. (2013). Influence of group embryo culture strategies on the blastocyst development and pregnancy outcome. *J. Assist. Reprod. Genet.* 30 63–68. 10.1007/s10815-012-9892-x 23239126 PMC3553343

[B22] Weimar C. H. Macklon N. S. Post U. E. Brosens J. J. Gellersen B. (2013). The motile and invasive capacity of human endometrial stromal cells: implications for normal and impaired reproductive function. *Hum. Reprod. Update* 19 542–557. 10.1093/humupd/dmt025 23827985

[B23] Wintner E. M. Hershko-Klement A. Tzadikevitch K. Ghetler Y. Gonen O. Wintner O. (2017). Does the transfer of a poor quality embryo together with a good quality embryo affect the In Vitro Fertilization (IVF) outcome? *J. Ovarian Res.* 10:2. 10.1186/s13048-016-0297-9 28086935 PMC5237322

[B24] Zeng Y. Hesketh T. (2016). The effects of China’s universal two-child policy. *Lancet* 388 1930–1938. 10.1016/S0140-6736(16)31405-2 27751400 PMC5944611

[B25] Zhu L. Xi Q. Zhang H. Li Y. Ai J. Jin L. (2013). Blastocyst culture and cryopreservation to optimize clinical outcomes of warming cycles. *Reprod. Biomed. Online* 27 154–160. 10.1016/j.rbmo.2013.04.006 23769665

